# Plasticity and the evolution of group-level regulation of cellular differentiation in the volvocine algae

**DOI:** 10.1098/rspb.2024.2477

**Published:** 2025-03-19

**Authors:** Dinah R. Davison, Aurora M. Nedelcu, Onyi De Andre Eneji, Richard E. Michod

**Affiliations:** ^1^ Department of Ecology and Evolutionary Biology, University of Arizona, Tucson, AZ, USA; ^2^ Kansas State University Division of Biology, Manhattan, KS, USA; ^3^ University of New Brunswick Department of Biology, Fredericton, New Brunswick, Canada; ^4^ Health Sciences, University of Arizona, Tucson, AZ, USA

**Keywords:** plasticity, individuality, multilevel selection, volvocine algae, cellular differentiation, evolution of multicellularity

## Abstract

During the evolution of multicellularity, the unit of selection transitions from single cells to integrated multicellular cell groups, necessitating the evolution of group-level traits such as somatic differentiation. However, the processes involved in this change in units of selection are poorly understood. We propose that the evolution of soma in the volvocine algae included an intermediate step involving the plastic development of somatic-like cells. We show that *Eudorina elegans,* a multicellular volvocine algae species previously thought to be undifferentiated, can develop somatic-like cells following environmental stress (i.e. cold shock). These cells resemble obligate soma in closely related species. We find that somatic-like cells can differentiate directly from cold-shocked cells. This differentiation is a cell-level trait, and the differentiated colony phenotype is a cross-level by-product of cell-level processes. The offspring of cold-shocked colonies also develop somatic-like cells. Since these cells were not directly exposed to the stressor, their differentiation was regulated during group development. Consequently, they are a true group-level trait and not a by-product of cell-level traits. We argue that group-level traits, such as obligate somatic differentiation, can originate through plasticity and that cross-level by-products may be an intermediate step in the evolution of group-level traits.

## 1. Introduction

Evolutionary transitions in individuality occur when groups of individuals evolve into a new kind of individual [1–3]. This process is fundamental to the evolution of multicellularity, during which the primary unit of selection and adaptation transitions from the cell to a group of cells, the multicellular individual. This transition requires the evolution of true group-level traits [2,4], which are properties of the group that arise from the developmental processes that create the group. Since they emerge through developmental processes that require interactions among multiple cells, true group-level traits cannot be defined in terms of cell-level traits. Our definition of true group-level traits is in line with other authors, though we primarily emphasize processes that create group-level traits rather than focusing on trait heritability [5–8] or fitness [9,10]. Identifying true group-level traits and how they arise is central to the study of evolutionary transitions in individuality and the origins of multicellular life. However, determining whether a trait is a true group-level trait can be challenging, as traits can appear to be properties of the group (and even benefit the group) but instead be aggregative by-products of the properties of the lower-level units. Aggregative by-products are not true group-level adaptations; instead, they reflect the aggregative properties of the lower-level units in the group. Such traits can be considered cross-level by-products because traits that appear to be properties of the group are actually side effects of cell traits [11].

Group-level traits can promote selection acting at the group level and/or suppress selection acting on lower-level units [2]. For example, cellular differentiation in multicellular organisms is a true group-level trait because it is regulated during development. Specialized somatic and reproductive cells contribute to different fitness components in a multicellular individual. They are less likely to survive and reproduce outside of the context of the group and sterile somatic cells cannot recreate the group. Groups with cellular specialization become indivisible and, thus, a new kind of individual. Consequently, during the evolution of multicellular individuality and cellular differentiation, selection transitions from acting on cells to acting on the group as new group-level traits evolve.

How group-level traits emerge from cell-level traits is a fundamental problem in multilevel selection theory [5,11]. In the case of multicellularity, true group-level traits emerge from the developmental processes that create the group and can evolve through the co-option of cell-level traits [4]. This transition requires a shift in the regulation of a trait from being primarily under cell-level control to being under the purview of the group. For instance, consider the regulation of cellular differentiation. Unicellular organisms can regulate their cellular state in response to internal and external cues. In multicellular organisms with specialized cell types, cell fate is specified during development, and the differentiated group phenotype is inherited when the organism reproduces. Therefore, the regulation of cellular differentiation is initially a cell-level trait and comes under the control of the cell group as multicellular development evolves. However, how this change in control can happen is not well understood.

One possibility is that phenotypic plasticity may mediate the transition from cell-level regulation to group-level regulation of cellular state. Phenotypic plasticity allows organisms to respond to their environment by altering how their phenotype develops [12], as is seen in environmentally induced changes in a unicellular organism’s differentiated state. In multicellular organisms, changes in cell interactions and developmental processes can also occur in response to an environmental cue; such plastic responses are group-level traits. Moreover, plastic responses to the environment that cause changes in group-level regulation involve both environmental responses and developmental responses, as development can change in response to an environmental cue [13]. Since plasticity can involve cell- or group-level responses to the environment, it could potentially bridge the evolutionary gap between cell- and group-level traits.

Here, we ask how the control of cellular differentiation changes from being an environmentally regulated cell-level trait to a developmentally regulated group-level trait. We hypothesize that phenotypic plasticity mediated the transition from a cell-level trait to a group-level trait during the origin of somatic differentiation [13–16]. To address how group-level traits evolve from cell-level traits and explore the role of phenotypic plasticity in this transition, we use the volvocine green algae as an experimental model system.

### (a) Model system: the volvocine green algae

**Figure 1.**
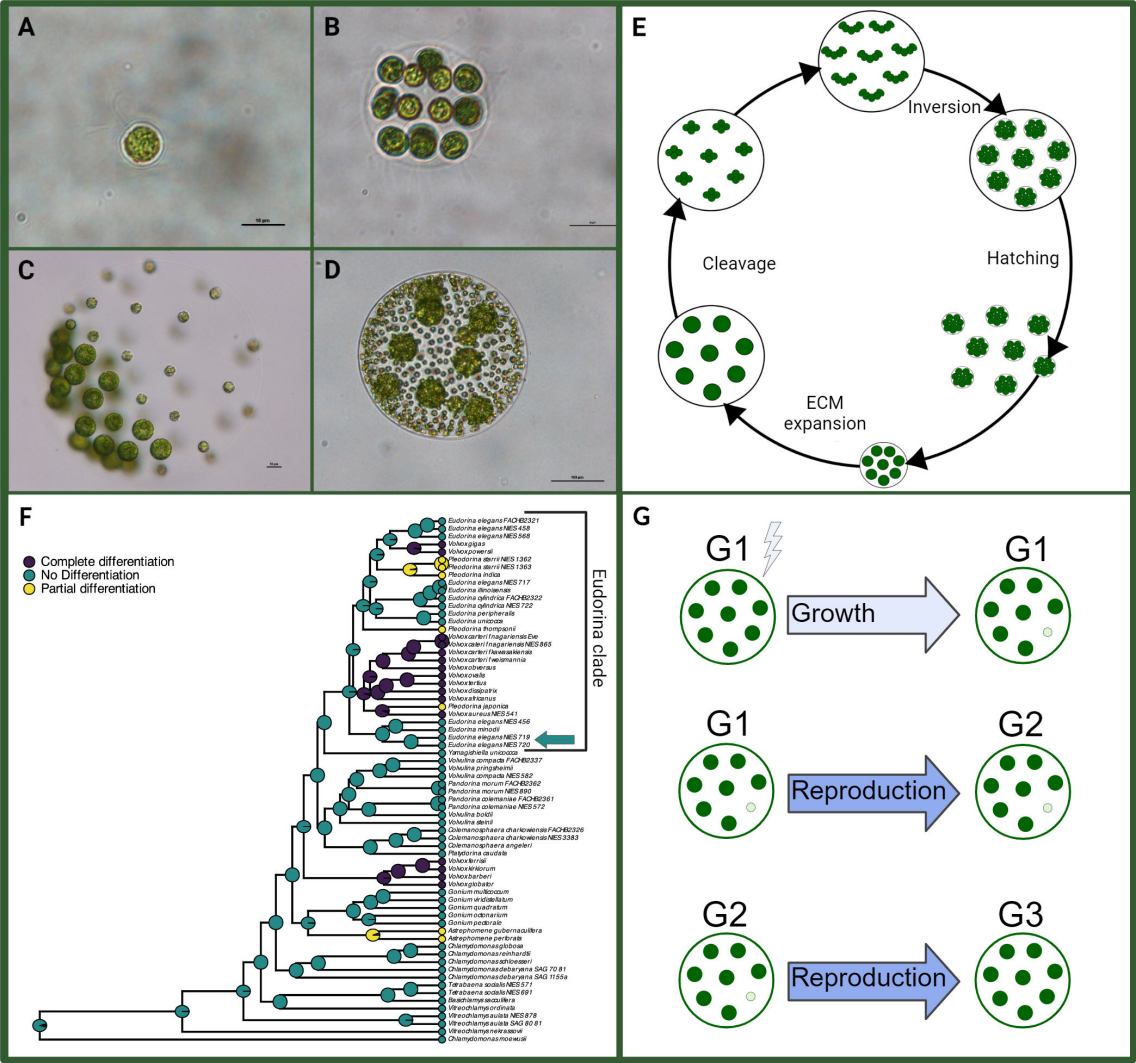
Representative volvocine algae species and the *Eudorina* life cycle. (A) Unicellular *Chlamydomonas reinhardtii* CC124. (B) Undifferentiated *Eudorina elegans* UTEX 1201. (C) Soma-differentiated *Pleodorina californica* UTEX 198. (D) Germ-soma differentiated *Volvox carteri* Eve. (E) Asexual life cycle of *E. elegans*. During the reproductive phase, all cells in a colony undergo several cleavage divisions followed by inversion, giving rise to offspring colonies. The offspring colonies then hatch out of the parent colony. The deposition of extracellular matrix causes colonies to expand in size without the addition of new cells. (F) A volvocine phylogeny showing an ancestral state reconstruction of cellular differentiation. Pie charts at the nodes represent the posterior probabilities based on stochastic character mapping. Teal nodes and tips correspond to undifferentiated species and ancestral populations, yellow refers to obligate somatic differentiation, and purple represents obligate germ and somatic cells. The Eudorina clade is denoted with the bracket. The teal arrow points to the region of the phylogeny containing *E. elegans* UTEX 1201 (inferred from [17], which found that *E. elegans* UTEX 1201 is sister to *E. elegans* NIES 719). Figure modified from [18] with permission. (G) A summary of the three generations examined in this experiment. G1 is cold-shocked and the differentiated phenotype (when present, shown through the presence of a smaller cell) occurs as a direct response to the stress. G2 colonies are the offspring of G1, and the differentiated phenotype (when present) arises during development. G3 are the offspring of G2. Figure modified from [13] with permission. Created with BioRender.com/b07d532.

The volvocine green algae are a monophyletic clade that contains unicellular forms (e.g. *Chlamydomonas*; figure 1A), undifferentiated multicellular species (e.g. *Eudorina*; figure 1B; all cells grow and then reproduce), and multicellular species with one or two specialized cell types, including somatic cells (e.g. *Pleodorina* and *Volvox*; figure 1C,D) [19,20]. Somatic cells are small, bi-flagellated, and do not reproduce; instead, they contribute to the organism’s motility and survival. Species with obligate somatic cells always develop terminally differentiated sterile somatic cells, regardless of the environment.

Somatic cells have evolved repeatedly in the Eudorina clade, a monophyletic group embedded within the broader volvocine green algae [21,22]. The Eudorina clade is composed of members of the polyphyletic genera *Volvox* [23] (figure 1D), *Pleodorina* [24] (figure 1C) and *Eudorina* [25] (figure 1B; [17,18,24–27]; figure 1F). *Pleodorina* and *Eudorina* are typically distinguished from each other based on the presence or absence of obligate somatic cells (e.g. [18,24,27–30]) with Coleman [30] stating ‘the primary difference between *Pleodorina* and *Eudorina* is in the consistent presence in *Pleodorina* of a proportion of anterior cells in the colony that never undergo daughter colony formation.’

To investigate the intermediate stages in the transition to obligate somatic differentiation, we used the multicellular volvocine algae *Eudorina elegans* UTEX 1201 (hereafter referred to as ‘*E. elegans’). E. elegans* has been characterized as undifferentiated [17]. It is a member of a small clade of undifferentiated *Eudorina* species that form an outgroup to the rest of the Eudorina clade; phylogenetic reconstructions have found that this outgroup likely never had an obligately differentiated ancestor [17,18,27]. *E. elegans* colonies are spherical and typically consist of 4−32 bi-flagellated, undifferentiated cells embedded in extracellular matrix (figure 1B). Each undifferentiated cell in a colony grows and divides to give rise to offspring colonies of 4−32 cells (figure 1E).

We exposed *E. elegans* to an ecologically relevant, transient environmental stressor (cold shock) and observed that some colonies had cells that appeared morphologically consistent with the somatic cells of other species. We frequently observed these cells in cold-shocked colonies and their offspring and observed that they were also present at lower frequencies in control conditions. We determined whether these somatic-like cells are similar to developmentally controlled somatic cells in obligately differentiated volvocine species. We then asked if the stress-induced differentiation of the somatic-like cells is a property of the cell and under environmental control, a property of the group and developmentally regulated in response to the environment, or both.

## 2. Methods

### (a) Induction of somatic-like cells over multiple generations

To determine whether somatic-like cells develop in response to cold shock, we grew *E. elegans* in 250 ml of standard Volvox media (SVM) in triplicate on bubblers at 25°C with a 16:8 h light:dark cycle. We inoculated one control flask and one treatment flask from each culture and cold-shocked the three treatment flasks by placing them in an ice bath at 1°C for 2 h before returning them to the bubblers.

To assess the development of somatic cells over multiple generations following cold shock, we imaged the cold-shocked colonies (which we refer to as generation 1 (G1)) and their offspring (generation 2 (G2); figure 1G). We did the same for two generations of controls. We examined 1 ml of culture daily for 4 days to determine when colonies reproduced. We imaged 50 expanding or expanded adult colonies (figure 1E) from each cold-treated and control flask for G1 and again for G2 on slides on a Leica DM500 light microscope. We recorded the number of somatic and reproductive cells.

### (b) Inheritance of somatic-like cells

We tracked the development of somatic-like cells over three generations to determine whether the somatic-like cell phenotype is plastic or due to recurrent mutations. We grew two flasks of *E. elegans* on a shaker. We then chose 48 unexpanded juvenile colonies (figure 1E) without visible somatic-like cells from the flasks. Each colony was transferred to an Eppendorf tube with 40 µl of SVM. Half of the Eppendorf tubes were placed in an ice bath for 2 h before the contents of all the Eppendorf tubes were transferred into separate wells in 24-well plates containing fresh media. We imaged all G1 colonies, transferred each G2 offspring to separate wells, and imaged each G2 colony and their generation 3 (G3) offspring. Imaging was conducted with a Nikon Eclipse Ti-E. The data results from two replicate experiments. A summary of the three generations is shown in figure 1F.

Our final sample sizes were affected by colony survival, successful transfer to or from wells, and the number of surviving reproductive cells (and, therefore, the number of offspring) present in G1 and G2. We collected data from 12 cold-shocked G1 colonies, 19 control G1 colonies, 72 cold-shocked G2 colonies, 199 control G2 colonies, 661 cold-shocked G3 colonies and 1267 control G3 colonies.

Each time we imaged a colony, we recorded the number of large (reproductive) and small (somatic-like) cells. Since we tracked the differentiation state of each colony and that of their offspring, we were able to characterize whether the differentiated phenotype was plastic or inherited. Specifically, we determined whether differentiated G2 colonies had more differentiated G3 offspring than undifferentiated G2 colonies.

To further examine whether the somatic-like cell phenotype can be inherited in G3 and determine what proportion of cells and colonies die following cold shock, we grew one flask of *E. elegans* on a shaker in controlled conditions. We then placed 1 ml of culture (figure 1E) in 6 Eppendorf tubes. We cold-shocked three tubes in an ice bath for 2 h before transferring the contents of all 6 tubes to fresh media in separate wells of a 6-well plate. Only one replicate was used during this experiment.

We imaged approximately 100 G1 cold-shocked colonies, 100 of their G2 offspring, and 100 G3 grand-offspring (figure 1G). We did the same for three generations of controls. We imaged colonies using brightfield and fluorescence microscopy on a Nikon Eclipse Ti-E. Chlorophyll autofluorescence was used as a proxy to determine whether the cell was likely alive. This basic experimental set-up was also used for subsequent experiments described below. A visual summary of this experiment is available in electronic supplementary material, methods S1.

### (c) Separation of single cells

To determine whether somatic-like cells can develop without cell–cell interactions, we separated two single cells from 20 unexpanded (figure 1E) undifferentiated colonies using needles to break apart the colonies. We placed each cell into a separate Eppendorf tube with 40 µl of SVM. Half were cold-shocked in an ice bath for 2 h, and all 40 cells were plated on separate 1% agar (in SVM) plates to prevent cell–cell communication and ensure that cells could be seen and tracked. Each plate was imaged using a Nikon Eclipse Ti-E microscope 3−6 days after the treatment. The experiment was replicated with an additional 40 cells from 20 unexpanded colonies.

### (d) Comparison to obligate somatic cells

We compared *E. elegans* somatic-like cells to the somatic cells of other species. For each of the following experiments, we cold-shocked colonies and grew them in well plates following the above methods. Somatic cells in obligately differentiated species are living cells. To determine if plastic somatic-like cells are also alive, we stained G1 and 2 expanded adult colonies with the vital stain fluorescein diacetate (FDA; 1 mM FDA stock solution in dimethyl sulfoxide). FDA is a non-fluorescent compound broken by cellular non-specific enzymes into a fluorescing fluorescein and has been used to assess *Chlamydomonas* viability [31–34]. We cold-shocked colonies and grew them in well plates following the above methods. We incubated G1 and G2 colonies for 20 min at 25°C in the dark at a concentration of 25 µm FDA following [34]. As a positive control, we killed colonies with 2% glutaraldehyde (commonly used to fix green algae [35]). After staining, we imaged colonies with a Nikon Ti-E microscope and used a GFP filter for fluorescence microscopy.

Somatic cells in obligately differentiated species have flagella whose movement contributes to colony motility. To determine if somatic-like cells have functional flagella, we cold-shocked colonies and grew them in well plates following the above methods. We captured images and videos of G1 and G2 colonies at 100× on the Nikon Eclipse Ti-E microscope.

To determine if somatic-like cells are significantly smaller than reproductive cells and the same size as somatic cells in other species, we measured reproductive and somatic-like cells in expanded (figure 1E) *E. elegans* colonies, reproductive and somatic cells in *Pleodorina starrii* NIES 1362 and the somatic cells in *Volvox carteri* Eve. We grew two flasks with *P. starrii* and *V. carteri* on a shaker. We repeatedly withdrew 10 µl of media from the flask and imaged the expanded adult colonies on a slide with the Nikon Eclipse Ti-E microscope.

We measured cell sizes using ImageJ [36]. We traced the perimeter of each cell in a colony using the circle tool in ImageJ, calibrated our measurements using the scale bar from each image and obtained the minimum diameter, maximum diameter and area of each cell.

Somatic cells in obligately differentiated species are terminally differentiated and do not give rise to the next generation. We cold-shocked colonies and grew them in plates using the above-mentioned methods to determine if somatic-like cells reproduce, we tracked colonies in well plates to determine if somatic-like cells reproduce. After the offspring of cold-shocked G1 colonies hatched, the G1 shells composed of extracellular matrix and somatic-like cells were transferred to new wells. The wells containing shells were monitored for 5 days to determine if any somatic-like cells reproduced. The same procedure was followed for the G2 colonies. Additionally, 12 control colonies were transferred to separate wells, and their offspring remained in the wells after reproduction. We examined these wells after 5 days and recorded whether viable colonies were still present to ensure that the growth conditions did not inhibit reproduction.

### (e) Statistical analysis

We performed statistical analyses using Python’s SciPy package and the R v.4.0.4 packages stats, MASS, moments and fitdistrplus. Statistical tests were chosen based on data type, sample size, data distribution and experimental design. We carried out four main types of statistical tests in our analyses.

We used the Welch unequal variances *t*‐test to compare the mean cell sizes of two groups when the data were approximately normally distributed and the variances were unequal. We employed the Wilcoxon rank-sum test to compare the number of somatic-like cells per differentiated colony between treatment groups. This test was applied when the data were not normally distributed or the sample sizes were unequal. We performed Pearson’s chi-squared test to analyse associations between treatment conditions and the presence of somatic cells. Finally, we used Fisher’s exact test to compare the proportions of differentiated colonies between two treatment conditions or generations.

## 3. Results

### (a) Cold shock results in the development of somatic-like cells in generations 1 and 2

**Figure 2.**
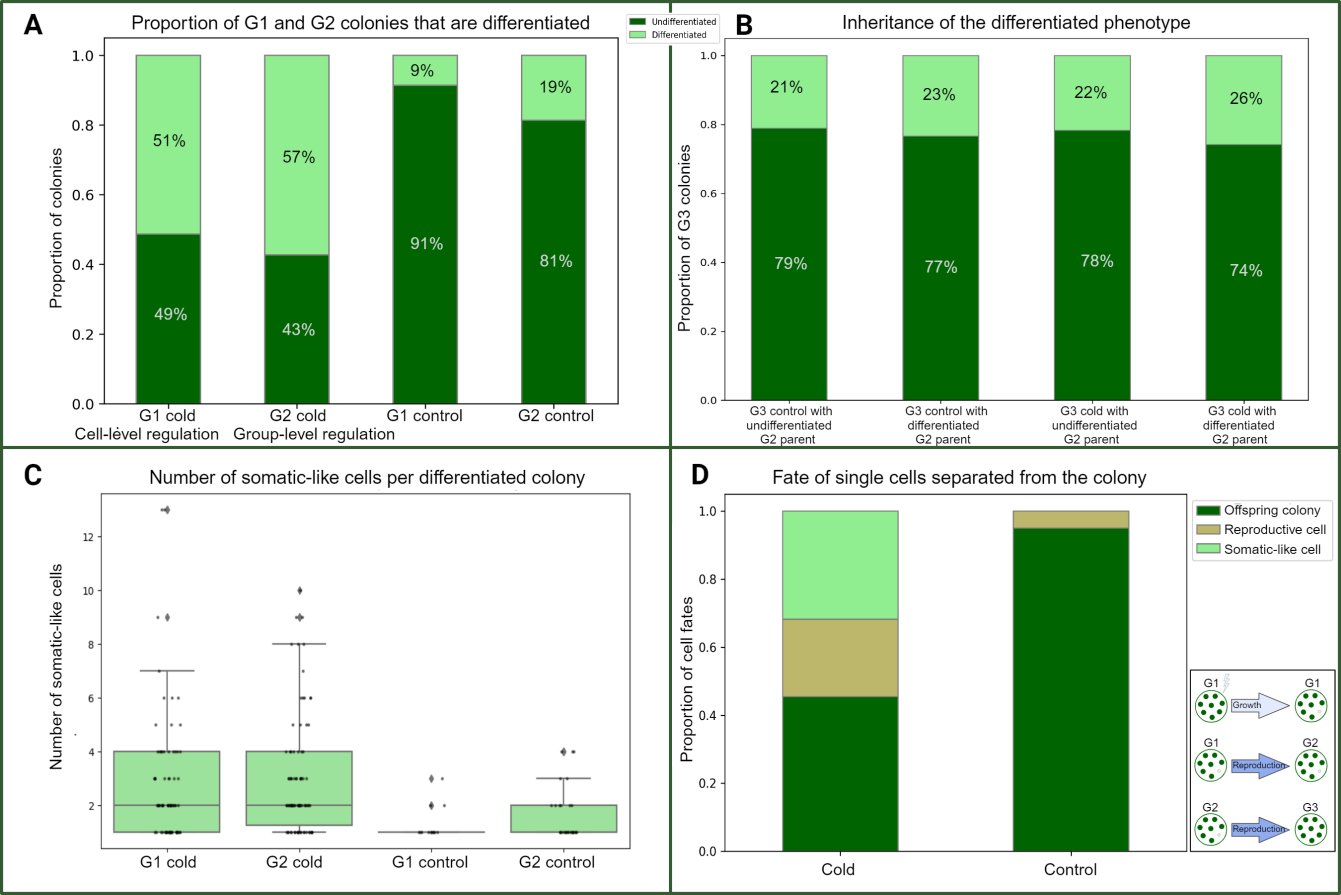
Somatic-like cells develop in response to environmental stress. (A) More cold-treatment G1 and G2 colonies have somatic-like cells than do corresponding controls. The proportion of colonies with (light green) and without (dark green) somatic-like cells is shown for G1 and G2 cold-shocked and control treatments. The percentage of differentiated or undifferentiated colonies is shown on the bars. (B) The differentiated phenotype is not inherited. The number of colonies with (light green) and without (dark green) somatic-like cells is shown for G3 cold-treatment and control colonies, grouped by whether their G2 parent had somatic-like cells. The percentage of differentiated or undifferentiated colonies is shown on the bars. (C) The number of somatic-like cells per differentiated G1 and G2 colony. Box plots show the proportion of somatic-like cells per differentiated colony. The dots show the underlying data distribution. (D) Cells can become somatic-like in response to cold shock outside the colony context. The fates of single cells were tracked following their separation from the colony. The bars show the proportion of cold-shocked and control single cells that became somatic-like (light green), reproduced and gave rise to an offspring colony (dark green) or were large reproductive cells but did not reproduce and were most likely dead (khaki). A summary of the three generations from figure 1F is reproduced here for reference. Bar charts were generated in Python. Created with BioRender.com/b23e901.

To address whether environmental stress can induce the development of somatic-like cells in *E. elegans*, we cold-shocked colonies and monitored the development of somatic-like cells in G1 colonies and their G2 offspring (see §2). We found that somatic-like cells differentiated in the G1 cold-shocked colonies and the G2 offspring of cold-shocked colonies at higher frequencies than in controls (table 1 and and figure 2). Even though members of the *Eudorina* genus are typically characterized as lacking cell differentiation, somatic-like cells were also present in some control colonies (albeit at lower frequencies than after cold shock; table 1 and figure 2 ).

**Table 1.** The proportion of living colonies with somatic-like cells and the mean (and s.d. of the mean) number of somatic-like cells per differentiated colony..

treatment	generation	level of regulation of differentiation	number of colonies	number of colonies with somatic-like cells	proportion of colonies with somatic-like cells	mean (s.d.) number of somatic-like cells per differentiated colony
cold	G1	cell-level	150	77	51.33%	2.75 (2.36)
cold	G2	group-level	150	86	57.33%	2.97 (2.16)
control	G1	unclear	150	13	8.67%	1.31 (0.63)
control	G2	unclear	150	28	18.67%	1.68 (1.02)

The cells in G1 colonies experienced cold shock, and consequently, somatic-like cell development occurred in direct response to the environment. The proportion of colonies with somatic-like cells was significantly higher in G1 cold-shocked colonies than in G1 controls (Fisher’s exact test for count data, two-sided, *p* = 1.81 × 10^−16^, *n* = 300; table 1 and figure 2). In total, 77 of the 150 G1 colonies had one or more somatic-like cells (51.3%). In contrast, only 13 of the 150 G1 control colonies had one or more somatic-like cells (8.7%).

The somatic-like cells in G2 colonies did not directly experience cold shock. Instead, G2 colonies developed from the undifferentiated cells of parent colonies that experienced cold shock. The proportion of colonies with somatic-like cells was also significantly higher in the G2 offspring of cold-shocked colonies than in controls: 86 of 150 (57.3%) offspring of cold-shocked colonies had somatic-like cells, compared to only 28 of 150 (18.7%) G2 controls (Fisher’s exact test for count data, two-sided, *p* = 5.38 × 10^−12^, *n* = 300; table 1 and figure 2A).

The proportions of differentiated G1 and G2 cold-treated colonies were not significantly different (Fisher’s exact test for count data, two-sided, *p* = 0.35). However, the proportion of differentiated G2 control colonies was significantly higher than that of G1 control colonies (Fisher’s exact test for count data, two-sided, *p* = 0.018). Since the number of G2 colonies in each flask is higher than in G1 due to G1 reproducing, their development may have been induced by the stress associated with higher colony density. The replicates were not significantly different (chi-squared tests for independence; *p* > 0.05); full results from replicate comparisons are available in electronic supplementary material, results S3.

The number of somatic-like cells per differentiated colony was highest in cold-shocked G1 colonies and their G2 offspring (table 1). Specifically, cold-shocked G1 colonies (*n* = 77) had significantly more somatic-like cells per differentiated colony than G1 controls (*n* = 13; 2.75 versus 1.31 somatic-like cells; Wilcoxon rank-sum test, two-sided, *W* = 2.80, *p* = 0.0051). The G2 offspring of cold-shocked colonies (*n* = 86) also had significantly more somatic-like cells per differentiated colony than differentiated G2 controls (*n* = 28; 2.97 versus 1.68 somatic-like cells; Wilcoxon rank-sum test, *W* = 3.24, *p* = 0.0012). These data are shown in table 1 and figure 2C.

**Figure 3.**
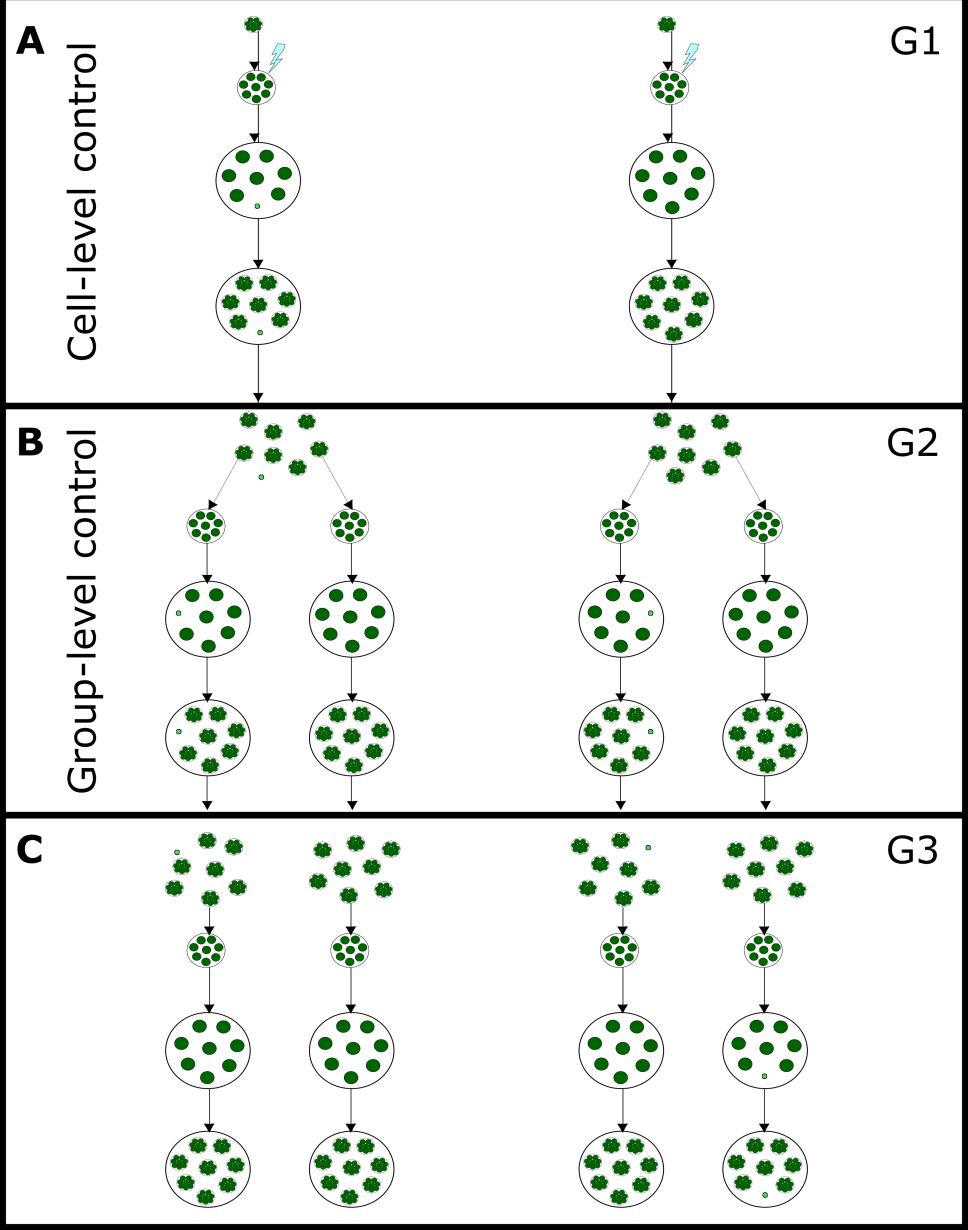
Summary of the plastic responses in response to cold stress. (A) The development of two G1 colonies following cold shock. All the undifferentiated cells (dark green) reproduce. In the left colony, one cell (small and light green) becomes somatic-like and does not reproduce. Since the environment directly induces G1 somatic-like cells, their differentiation is under cell-level control. (B) The development of four of the G2 offspring of the G1 colonies. Some of the G2 offspring colonies develop somatic-like cells (small and light green). Colonies with somatic-like cells can develop from either differentiated or undifferentiated G1 parents. Since G2 somatic-like cells arise during development in the offspring of cold-shocked colonies, their development is under group-level control. (C) Four of the G3 grand-offspring of G1 colonies. Three of the colonies are undifferentiated and one has a somatic-like cell. The differentiated colony was chosen randomly; the presence or absence of somatic cells in G2 does not affect the likelihood that G3 offspring will be differentiated.

Since G1 somatic-like cells differentiated directly from cells that experienced the cold shock, this change is likely a cell-level response to environmental stress. In contrast, G2 colonies developed from cells in G1 colonies that reproduced after they experienced cold shock. Thus, somatic-like cells in G2 result from developmental changes and can be considered a group-level response. We summarize these results in figure 3. The dataset and corresponding images are available on Dryad as electronic supplementary material, S1 and S5, respectively.

### (b) The stress-induced differentiated phenotype is not inherited

Since the proportion of differentiated colonies was higher in G1 cold-shocked colonies and their G2 offspring than in controls, we examined whether the differentiated phenotype was caused by plasticity or recurrent, cold-induced mutations. We followed the individual offspring of each G2 colony and compared the proportion of colonies with somatic-like cells produced by differentiated and undifferentiated parent colonies.

The differentiation status of G2 colonies did not affect the likelihood that their G3 offspring would be differentiated. Differentiated cold-treatment G2 colonies did not produce a different proportion of differentiated offspring than undifferentiated cold-treatment G2 colonies (Pearson’s chi-squared test, *X*² = 1.87, *p* = 0.2, *n* = 661). Similarly, the proportion of G3 control colonies with somatic-like cells was not affected by whether the G2 parent was differentiated (Pearson’s chi-squared test, *X*² = 0.50, *p* = 0.5, *n* = 1267). These results are shown in figure 2B.

The proportion of colonies with somatic-like cells was not significantly different between the G3 cold-treatment colonies and G3 controls (Fisher’s exact test for count data, two-sided, *p* = 0.07, *n* = 203). Furthermore, the cold-treatment and control G3 colonies did have significantly different proportions of somatic-like cells per colony (Wilcoxon rank-sum test, two-sided, W = 4669.5, *p* = 0.051, *n* = 203; table 1 and figure 2B). The dataset is available on Dryad as electronic supplementary material, S2.

In a separate experiment, we cold-shocked colonies and imaged them for three generations. The proportion of colonies with somatic-like cells was not significantly different between G3 cold-treatment colonies and G3 controls (Fisher’s exact test for count data, two-sided, *p* = 0.07, *n* = 203). Nine of 102 (8.8%) G3 cold-treatment colonies had somatic-like cells, compared to 18 of 101 (17.8%) controls. Full results from this experiment are available in electronic supplementary material, results S2, and the dataset is available on Dryad as electronic supplementary material, S3.

Taken together, these data illustrate that the differentiated phenotype is not inherited. Since the frequency of somatic-like cells in the G3 grand-offspring of cold-shocked colonies did not differ from controls and G2 colonies with somatic-like cells were not more likely to produce differentiated offspring, somatic-like cells are most likely a plastic developmental response to the environment and not due to heritable recurrent mutations.

### (c) Generation 1 cold-shocked cells can differentiate into somatic-like cells outside the colony

The somatic-like cells observed in G1 colonies differentiated in direct response to cold stress, suggesting they are under cell-level control. However, G1 somatic-like cell differentiation could potentially be affected by interactions among cells, which would make them a group-level trait. To address whether the plastic differentiation of G1 somatic-like cells is under cell-level or group-level control, we removed 80 individual cells from colonies, cold-shocked half of them and monitored their fate and ability to reproduce on separate agar plates. Twenty-two of the cold-shocked cells were successfully observed. Ten of those cells went on to divide and develop into colonies, five remained the size of reproductive cells but did not reproduce and were most likely dead and seven were small and somatic-like and did not reproduce. Twenty of the control cells were observed on the plates. Nineteen of these cells divided and gave rise to the next generation, while one remained the size of a reproductive cell but did not reproduce and was most likely dead. The proportion of cell types differed significantly between the control and cold-shocked groups (Pearson’s chi-squared test, *X*^2^ = 9.83, *df* = 1, *p* = 0.002, *n* = 42). The small size and loss of reproductive ability of the cold-shocked cells indicate that the expression of the somatic-like phenotype in G1 is not due to cell interactions. The dataset is available on Dryad as electronic supplementary material, S6.

### (d) Plastic somatic-like cells share similar characteristics with obligate somatic cells

Somatic cells in obligately differentiated volvocine algae species are characterized as small, living, flagellated cells that do not reproduce. To determine whether somatic-like cells are consistent with the obligate somatic cells in other species, we examined the characteristics of G1 and G2 somatic-like cells.

**Figure 4.**
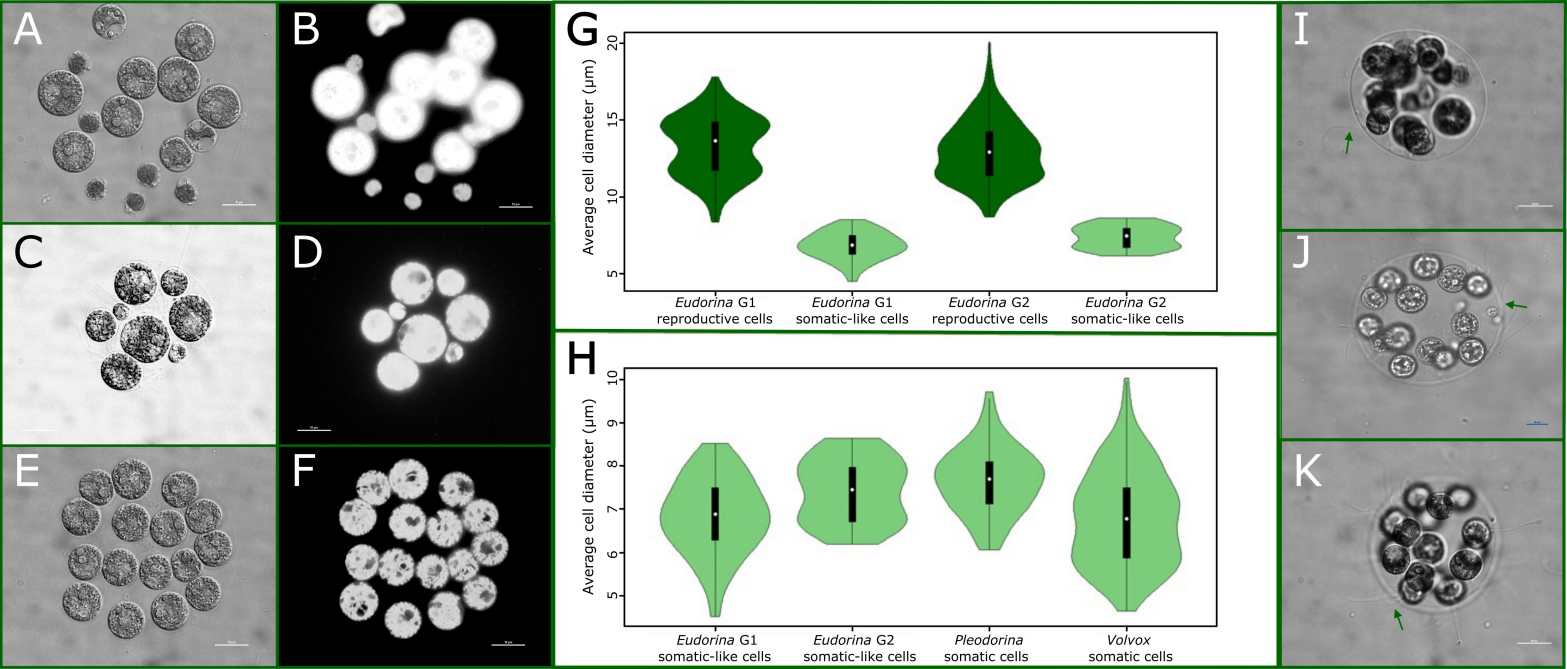
Plastic somatic-like cells are similar to the obligate somatic cells of other species. (A) Brightfield image of cold-shocked G1 *E. elegans*. (B) Cold-shocked G1 stained with FDA. Both the reproductive and somatic-like cells fluoresce, indicating they are alive. (C) Brightfield image of the G2 offspring of a cold-shocked colony. (D) A G2 cold-treatment colony stained with FDA. Both the reproductive and somatic-like cells fluoresce, indicating they are alive. (E) Brightfield image of a control colony. (F) A control colony stained with FDA. All cells fluoresce, indicating that they are alive. (G) The average cell diameters of G1 and G2 somatic-like cells are significantly different. Both G1 and G2 somatic-like cells are significantly smaller than reproductive cells. (H) The average cell diameters of *E. elegans* G1 and G2 somatic-like cells, *P. starrii* NIES 1362 somatic cells and *V. carteri* Eve somatic cells are shown. Somatic-like cell sizes do not differ significantly from the sizes of somatic cells. (I) A brightfield image of cold-shocked G1 *E. elegans*. The flagella of a somatic-like cell are visible. (J) A brightfield image of cold-shocked G2 *E. elegans*. The flagella of a somatic-like cell are visible. (K) A brightfield image of control *E. elegans*. The flagella of undifferentiated cells are visible.

We stained expanded G1 and G2 colonies with the vital stain FDA to determine if somatic-like cells are alive. We found that G1 cold-shocked colonies had fluorescent somatic-like cells and fluorescent reproductive cells, indicating that G1 somatic-like cells are most likely alive. The G2 offspring of cold-shocked colonies also had fluorescent somatic-like and reproductive cells, indicating that G2 somatic-like cells are also most likely alive. Representative stained colonies are shown in figure 4A–F. Cell and whole-colony mortality were observed in some cold-shocked colonies. Chloroplast autofluorescence was also observed in both generations’ living reproductive cells and somatic-like cells. As controls, both somatic and reproductive cells in *V. carteri* Eve and *P. californica* UTEX 809 fluoresced when stained with FDA and exhibited chlorophyll autofluorescence. As expected, cells in *E. elegans* colonies killed with 2% glutaraldehyde did not fluoresce.

Since somatic cells in the volvocine algae are characterized in part by their small size, we measured and compared the diameter of somatic-like, somatic and undifferentiated reproductive cells in *E. elegans*, *P. starrii* NIES 1362 and *V. carteri* Eve (figure 4G,H). *E. elegans* G1 and G2 somatic-like cells were significantly smaller than undifferentiated cells. Somatic-like cells in G1 had an average maximum diameter of 6.83 µm (*n* = 88). Somatic-like cells in G2 had an average diameter of 7.43 µm (*n* = 41). G1 somatic-like cells were significantly smaller than G2 somatic-like cells (Welch unequal variances *t*‐test, two-sided, *t* = −4.11, *df* = 94.29, *p* < 0.001). Both G1 (Welch unequal variances *t*‐test, two-sided, *t* = −37.75, *df* = 270.49, *p* < 2.2 × 10^−16^) and G2 (Welch unequal variances *t*‐test, two-sided, *t* = −34.179, *df* = 147.44, *p* < 2.2 × 10^−16^) somatic-like cells were significantly smaller than G1 and G2 undifferentiated cells (figure 4G).

To determine whether *E. elegans* somatic-like cells are similar in size to the soma of other species, we measured the diameters of *V. carteri* Eve and *P. starrii* NIES 1362. *Volvox* somatic cells had an average diameter of 6.88 ± 0.076 µm (*n* = 207), while *Pleodorina* somatic cells had an average diameter of 7.66 ± 0.098 µm (*n* = 60). The average diameter of *Volvox* somatic cells was not significantly different from that of *Eudorina* G1 somatic-like cells (*p* = 0.98), and the average diameter of *Pleodorina* somatic cells was not significantly different from the *Eudorina* G2 somatic-like cells (*p* = 0.58). Somatic cell diameters of *Eudorina* G1 and G2, *Pleodorina* and *Volvox* are shown in figure 4H. Complete comparisons are available in electronic supplementary material, results S4, and the dataset is available on Dryad as electronic supplementary material S4.

We observed flagella on G1 and G2 somatic-like and undifferentiated cells (figure 4I–K). These flagella were observed beating (see example electronic supplementary material, videos S5 and S6). Not all reproductive and somatic-like cells had flagella, and we observed flagella falling off when the coverslip was applied. Cold shock can also cause flagellar shortening and loss in *Chlamydomonas* [37].

To confirm that somatic-like cells do not reproduce, we followed the fate of somatic-like cells remaining behind after offspring hatch from the parental colony. After 5 days of culturing, none of the 12 wells containing G1 shells and somatic-like cells had offspring colonies. Similarly, 11 of 12 wells inoculated with shells containing G2 somatic-like cells lacked offspring colonies. In contrast, all 12 wells inoculated with control colonies continued reproducing.

## 4. Discussion

### (a) Cold shock induces somatic-like cell development in two generations

This study addresses how a trait regulated at the cell level can become a developmentally regulated group-level trait during the evolution of multicellularity. To explore this question, we focus on somatic specialization. The evolution of somatic specialization—a group-level trait—is a key step in this transition because somatic cells can no longer directly reproduce and, therefore, render the group indivisible—an individual.

**Figure 5.**
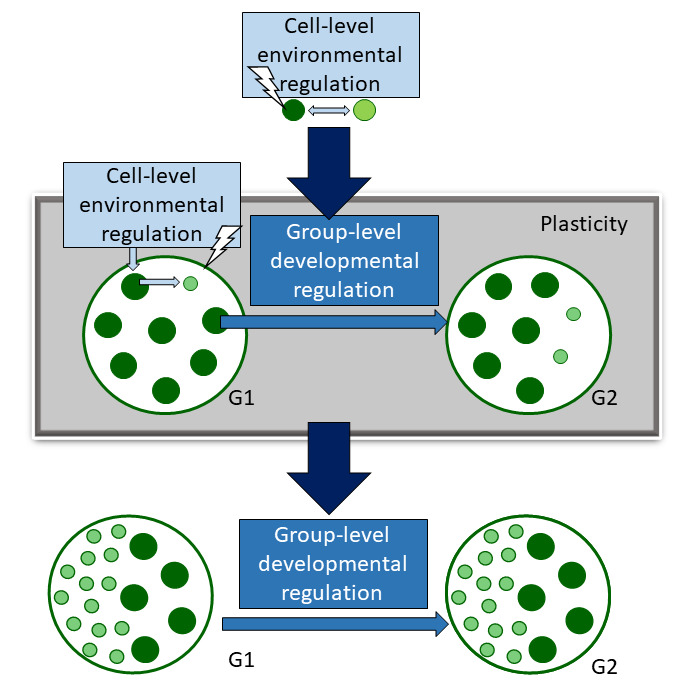
Plasticity may mediate the transition from cell-level, environmental regulation to group-level, developmental regulation of cellular differentiation. An environmental cue triggers a change in cellular state (the cessation of reproduction), shown by the change from dark green to light green in a unicellular individual. This is a cell-level response to the environment. The proposed intermediate stages discovered in this study are shown in the grey box. An environmental signal triggers a change in cellular state (from reproductive to somatic-like) in some cells in the group. This is a cell-level response. The environmental signal can also trigger a developmental change in some reproductive cells, as the development of the next generation is altered. This is a group-level response. Finally, we propose that environmental cell-level regulation was lost, and a change in the developmental signalling pathway led to the development of obligate somatic cells regardless of the environment. Figure modified from [13] with permission.

The Eudorina clade in the volvocine algae is composed of three polyphyletic genera, one of which (*Eudorina*) is described as undifferentiated. We focused on a *Eudorina* species that is hypothesized never to have had an obligately differentiated ancestor [17,18,27]. We found that *E. elegans* can develop plastic somatic-like cells following an ecologically relevant stressor. These cells are similar to the obligate somatic cells in other volvocine algae species—they are small, terminally differentiated cells that do not reproduce and have functional flagella. We observed plastic cell- and group-level regulation of cellular differentiation in *E. elegans* in response to environmental stress. We propose that the evolution of somatic differentiation in the Eudorina clade involved changes from cell-level environmental regulation to group-level developmental regulation cued by the environment, followed by group-level developmental regulation that occurs regardless of the environment (figure 5). We discuss different modes of regulation and their evolutionary implications below.

### (b) Cell-level and group-level control of cellular differentiation

The plastic somatic-like cells in G1 colonies are a cell-level response to environmental stress. The cells of G1 colonies directly experience the cold shock (figure 2). As a result, some cells cease to grow and do not reproduce, similar to the somatic cells of *Pleodorina* and *Volvox*. Finally, G1 somatic-like cells are a cell-level response because they can develop in direct response to the environment in the absence of interactions among cells (outside of the colony context). While *E. elegans* cells can differentiate outside the cell group, being in a colony could still modulate the response to cold shock. For example, the extracellular matrix of the colony could offer some protection against the deleterious effects of cold shock. The plasticity seen in G1 somatic-like cells might reflect the stress-response capabilities of the unicellular ancestor of the volvocine algae expressed in a multicellular context. G1 somatic-like cells may be in a permanent state of cell cycle arrest, which is a response to stress in both *Chlamydomonas* and the reproductive cells of *Volvox* [38,39]. This stress response was likely co-opted into the development of obligate somatic cells in species such as *V. carteri* since *regA*, a gene necessary for the development of *V. carteri* soma, is a homolog of *RLS1*, a gene required for the cessation of reproduction in *C. reinhardtii* in response to environmental stress [15,40–42].

In contrast, the expression of G2 somatic-like cells is likely a true group-level trait, as it is a multicellular developmental (at the group level) response to an environmental cue. While G2 colonies with somatic-like cells may be phenotypically similar to differentiated G1 colonies, the processes that give rise to the somatic-like cells must be distinct. Cells in G2 colonies developed from a cold-shocked parental cell and have not themselves experienced the cold shock (figures 1 and 3). Therefore, G2 somatic-like cells are a true group-level trait whose development was environmentally induced.

Somatic-like cells were also present in some control colonies. This observation indicates that stressors other than cold shock can induce somatic-like cell development. Consistent with this possibility, we found that 18.7% of G2 control colonies had somatic-like cells, while only 8.7% of G1 control colonies were differentiated (table 1 and figure 2A). Since each reproductive cell in a colony gives rise to an offspring colony, culture density increases substantially each generation. The increase in the proportion of differentiated colonies in control conditions may reflect the decrease in nutrients available to each colony in increasingly crowded flasks. Alternatively, somatic-like cell development in control colonies may result from developmental noise.

True group-level traits are characterized by group-level heritability. Since they are properties of the groups themselves (rather than by-products of the traits of lower-level units) and can be acted on by selection operating on groups, they must be inherited when groups reproduce [6–8]. The somatic-like differentiation we observed in *Eudorina* was a plastic response to the environment and, therefore, not heritable by virtue of being a plastic trait. However, the capacity to develop plastic somatic-like cells is likely heritable (though we do not directly address this here with our asexually reproducing strain). This possibility is in line with previous work, which has shown that plasticity (for a given trait) itself can be heritable and plastic responses can be shaped by selection (e.g. [43–46]).

The developmental processes that underpin plastic differentiation may be central to the transition to differentiated multicellularity. In *E. elegans*, the cell-level capability to differentiate is present and expressed in response to the environment. This response was observed in G1. The capacity for the group’s development to change in response to the environment, resulting in the development of G2 somatic cells, is also present. The cell-level capacity for individual cells to become somatic-like may have been a predecessor to the group-level capacity for development to regulate the production of somatic-like cells. This developmental regulation may have initially occurred in response to the environment and later become part of a canalized developmental programme (figure 5).

### (c) Cross-level by-products as a stage in the evolution of group-level traits

Since the development of G1 somatic-like cells is a cell-level response to environmental stress (figure 3 and 5), the G1 differentiated colony phenotype is a cross-level by-product of phenotypic plasticity at the cell level. Cross-level by-products are typically viewed as a challenge in the study of multilevel selection since they are by-products of lower-level traits that appear to be properties of the group [5,11,47]. While the development of cross-level by-products is controlled at the lower level, regulatory changes could result in this control being co-opted to the group level. In our system, cross-level by-products (such as G1 somatic-like cells) could have paved the way for the evolution of G2 somatic-like cells and obligate somatic cells, which are true group-level traits.

The evolution of cellular specialization requires changes to multicellular development; such development may utilize processes that previously existed at the cellular level, including cellular differentiation. Cell-level regulation of differentiation may have been placed in the group context (as is seen with the G1 somatic-like cells; figures 2, 3 and 5) during the evolution of complex multicellularity. Regulatory changes may then result in cellular specialization being part of group-level development, as seen in G2 somatic-like cells and obligate somatic cells in closely related species. Alternatively, the cell-level control of somatic-like cells may have evolved in parallel to (rather than before) the evolution of group-level control of somatic-like cells. More broadly, our results suggest that cross-level by-products could be a key intermediate step in the evolution of group-level traits.

### (d) Genetic accommodation and the transition from environmental to developmental regulation of cellular differentiation

We propose that the genetic accommodation of plastic somatic differentiation may have mediated the transition from environmental to developmental regulation of cellular differentiation (figure 5). In unicellular *Chlamydomonas* and G1 cold-shocked *E. elegans,* cellular differentiation is a cell-level trait cued by environmental conditions. In contrast, obligate somatic differentiation is developmentally regulated and has evolved repeatedly in the Eudorina clade. Plastic differentiation in *Eudorina* involves both environmental and developmental regulation and may be a stepping stone from environmental to developmental regulation.

Obligate somatic differentiation may therefore have repeatedly evolved via genetic accommodation. Genetic accommodation [12] occurs when the environmental regulation of a phenotype evolves; this evolution can take the form of increased or decreased plasticity. In the volvocine algae, somatic cell development may have been an ancestrally plastic response to the environment, with plasticity decreasing in lineages with obligate somatic cells [13]. However, the plastic response was not entirely lost, as obligately differentiated species such as *Pleodorina californica* exhibit increases in the proportion of somatic cells in response to environmental stress [48]. Additional research on other Eudorina clade species is needed to reconstruct the most likely ancestral state of the Eudorina clade and understand how plasticity has evolved during the transition to obligate differentiation.

### (e) Implications for the volvocine algae model system

The volvocine green algae are an important model system for studying the evolution of multicellularity and cell differentiation. Their utility stems in part from the existence of what is typically described as three categories of differentiation, which correspond to the three polyphyletic genera of the Eudorina clade: undifferentiated species (*Eudorina*), soma-differentiated species (*Pleodorina*) and germ-soma differentiated species (*Volvox*; figure 1B–D).

However, we found that differentiation in the Eudorina clade is more complicated. *E. elegans* can develop plastic somatic-like cells in response to the environment and consequently should not be classified as undifferentiated. Moreover, a smaller proportion of colonies have somatic-like cells in standard culture conditions. This may indicate that other aspects of culturing conditions (such as an increasing proportion of colonies in the cultures across generations) induce a stress response. Alternatively, *Eudorina* development may not be highly canalized, resulting in the stochastic development of somatic cells in the absence of an environmental stressor. It is unclear whether these cells result from cell-level or group-level processes.

Our findings are consistent with work by Goldstein [49] and Merchant [50]. Goldstein [49] described a *Eudorina* form he called hemidorina, which are likely colonies with somatic-like cells in a different *Eudorina* species. Similarly, Merchant [50] observed that a small proportion of *Eudorina* colonies exhibited somatic cells. Consequently, it appears likely that multiple *Eudorina* species may be capable of plastic somatic differentiation.

Moreover, species that are intermediate between *Pleodorina* and *Eudorina* have been described. The polyphyletic species *Pleodorina illinoisensis* Kofoid was originally assigned to the genus *Pleodorina* due to the presence of four small somatic cells in the anterior of the colony [51]. This species was reassigned as *Eudorina illinoisensis* (Kofoid) Pascher [52] reassigned this species as *Eudorina illinoisensis* (Kofoid) Pascher because he observed that the somatic cells occasionally divide and that the cells are arranged into tiers as was described for *E.* because its somatic cells are not always terminally differentiated (reviewed in [29]).

These observations suggest that plastic somatic differentiation should be considered a fourth category of differentiation in the volvocine algae and that the ancestor of the Eudorina clade may have had plastic somatic-like cells. Given that somatic-like cells possess the characteristics used to identify obligate somatic cells in a range of volvocine algae species, we suggest they should be considered plastic somatic cells. Studies reconstructing the evolution of cellular differentiation should therefore incorporate plastic differentiation. As discussed above, the repeated evolution of obligate somatic cells may reflect multiple instances of the genetic accommodation of plastic somatic cells rather than repeated de novo evolution of somatic differentiation.

The ecological relevance of small numbers of somatic cells is unclear and is an important area of future research. Since the species *E. illinoisensis* is polyphyletic [17] and is characterized by having a small number of somatic cells that are not always terminally differentiated [29,51], it seems likely that there is an adaptive benefit (or at least not a large cost) associated with having a small number of somatic cells. For example, developing small numbers of somatic-like cells may allow for increased resource allocation per reproductive cell. The likelihood that the developing colony will survive long enough to reproduce in harsh environments may be heightened if more developmental resources are invested in a limited number of reproductive cells at the expense of other cells. The cells that receive fewer resources may then become somatic-like. In addition to consuming fewer resources during development, these somatic-like cells may still produce extracellular matrix and contribute to colony motility, allowing the colony to swim near the top of the water column even when the reproductive cells are dividing and the colony is heavier. Consistent with this possibility, *P. californica* develops more somatic cells when grown in harsh environments [48]. Alternatively, plastic somatic cells may be a non-adaptive by-product of developmental-genetic processes occurring in response to environmental stress.

For example, developing small numbers of somatic-like cells may allow for increased resource allocation per reproductive cell. Reproductive cells in colonies that experience stressful environments may alter resource allocation during development to increase their chances of successfully developing into an adult colony and reproducing. The likelihood that the developing colony will survive long enough to reproduce may be heightened if more developmental resources are invested in a limited number of reproductive cells at the expense of other cells. The cells that receive fewer resources may then become somatic-like. In addition to consuming fewer resources during development, these somatic-like cells may still produce extracellular matrix and contribute to colony motility, allowing the colony to swim near the top of the water column even when the reproductive cells are dividing and the colony is heavier. Consistent with this possibility, *P. californica* develops more somatic cells when grown in harsh environments [48]. Alternatively, plastic somatic cells may be a non-adaptive by-product of developmental-genetic processes occurring in response to environmental stress.

Future work should examine additional *Eudorina* species to reconstruct the origins of somatic cells and determine if the developmental capacity for plastic differentiation is a defining characteristic of the genus. This work should also examine other Eudorina clade genera to characterize the evolution of developmental plasticity and determine which environmental conditions can regulate somatic development. Additionally, the genetic and developmental mechanisms of somatic-like cell development are unknown in both G1 and G2 and should be investigated. Lastly, experimental evolution could be used to characterize the evolution of obligate somatic cells and determine whether they can evolve via genetic accommodation.

## 5. Conclusions

The question of how group-level traits arise is central to the general theory of multilevel selection and the understanding of the evolution of multicellular life. Here, we focused on the group-level trait of somatic differentiation, a key step in the evolution of multicellularity. We show that plastic somatic-like cells in the volvocine algae species *E. elegans* could bridge the gap from environmental, cell-level control of cellular state to developmental, group-level control of differentiation. Plastic somatic-like cells in colonies that experienced an acute stressor developed because of cell-level processes; the colony phenotype of possessing somatic-like cells in response to stress is a cross-level by-product. We also found that the offspring of cold-shocked colonies develop somatic-like cells. Since these cells were not directly exposed to the stressor, their development was a true group-level trait induced by the environment experienced before the colony developed. We propose that the evolution of obligate somatic cells in closely related volvocine algae species may result from evolutionary changes in the regulation of this plastic response. We conclude that group-level traits can originate through plastic responses to the environment and that cross-level by-products may be a key intermediate step in the transition from cell-level to group-level control of cellular specialization.

## Data Availability

The data structure, code and data are all publicly available. The dataset titled ‘Plasticity and the evolution of group-level regulation of cellular differentiation in the volvocine algae’ is publicly accessible on Dryad [53]. The software files are publicly available on Zenodo [54]. Supplementary material is available online [55].

## Declaration of AI use

GitHub CoPilot and ChatGPT were used to troubleshoot coding problems and improve the readability of the code. Grammarly was used to edit the manuscript.
